# An Energy Focusing Flexible and Lightweight Acoustic Metamaterial for Enhanced Ultrasound Power Transfer

**DOI:** 10.1002/adma.202519545

**Published:** 2026-03-11

**Authors:** Hyung‐Suk Kwon, Ziqi Yu, Xiaopeng Li, Ercan M. Dede, Taehwa Lee

**Affiliations:** ^1^ Electronics Research Department Toyota Research Institute of North America Ann Arbor Michigan USA

**Keywords:** energy harvesting, flexible acoustic metamaterial, implantable medical devices, ultrasound power transfer, wireless battery charging

## Abstract

Ultrasound power transfer enables the wireless charging of implantable medical devices by utilizing an energy harvester to convert incoming ultrasound into electrical energy. Most harvesters rely on Lead Zirconate Titanate (PZT) crystals for energy conversion, but since PZTs are rigid and dense ceramics, scaling them up to harvest more energy produces heavier devices that are impractical for medical applications. This study presents a metamaterial‐enhanced ultrasound energy harvester (Meta‐UEH) that integrates a small PZT crystal with a locally resonant metamaterial, which concentrates ultrasound energy onto the PZT and thus improves its energy harvesting performance. The metamaterial is flexible, lightweight, and entirely passive, making it well‐suited for medical applications, while increasing the power of harvested electricity by more than double. Experimental and simulation results confirm that the Meta‐UEH achieves enhanced efficiency even under reverberation or deformation, benefiting from standing waves that form between transducers and the metamaterial, with harvested electrical power reaching up to 350% of that obtained without the metamaterial and standing wave. The underlying mechanisms behind the improvement are analyzed, revealing that the improvement is not specific to PZTs but can also be applied to other ultrasound‐electricity conversion methods, such as piezoelectric and triboelectric nanogenerators.

## Graphical abstract

A flexible and lightweight acoustic metamaterial composed of air cavities encased in polydimethylsiloxane (PDMS) is introduced to enhance ultrasound power transfer. The metamaterial leverages the strong reflectivity of air cavity boundaries to concentrate incoming ultrasound, thereby improving the performance of piezoelectric energy harvesters. Experimental results demonstrate a more than 100% increase in harvested power while maintaining robustness to deformation.

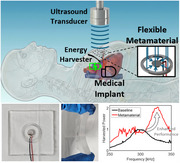

## 1 Introduction

Implantable medical devices (IMDs) are used for real‐time diagnostic and therapeutic applications such as brain stimulation, cardiac defibrillation, and programmable drug release [1, 2, 3]. IMDs are also expanding their functionality by pairing with wearable devices [4]. However, battery depletion without a means of recharging poses a life‐threatening safety risk for patients with IMDs. Moreover, the inability to recharge IMDs post‐implantation necessitates periodic surgeries to replace batteries, exposing patients to infection risk, higher costs, and time burdens, and limiting IMD functionality to tasks that do not require substantial power.

To overcome these limitations, researchers are investigating ultrasound power transfer (UPT) to charge IMDs remotely. In UPT, an external transducer transmits acoustic energy to an in vivo ultrasound energy harvester (UEH), where a mechanical‐electrical energy conversion element, typically Lead Zirconate Titanate (PZT), converts the incoming acoustic energy into electricity [5, 6]. To ensure safety and user comfort, UEHs for medical applications must be flexible, light, highly efficient, and use minimal electrical components to prevent failure. To satisfy these requirements, researchers are actively investigating various forms of UEHs. For example, some studies reported using PZT arrays instead of a bulky PZT to improve energy harvesting performance by collecting acoustic energy over a wide area while maintaining flexibility [7, 8]. Although this method resulted in flexible UEHs, it required excessive wiring of a large number of PZTs, thereby increasing the risk of electrical failures. Other studies proposed replacing rigid PZTs with more flexible acoustic‐electric energy conversion elements, such as piezoelectric nanogenerators (PENGs) and triboelectric nanogenerators (TENGs) [9, 10, 11, 12, 13]. Using PENGs and TENGs allowed UEHs to harvest acoustic energy over a wide area without compromising flexibility or requiring many electrical components. However, PENGs and TENGs are thin plates, through which most of the incoming ultrasound penetrates, and only a fraction of the acoustic energy is converted into electricity. Therefore, to capture sufficient acoustic energy, these systems relied on strong standing waves that form between an ultrasound transducer and a flat background parallel to each other, and their performance in reflectionless open space was never demonstrated. This validation approach does not accurately reflect real‐world scenarios, where backgrounds are typically non‐flat and not parallel to the transducer. As a result, their performance without the aid of strong standing waves should be measured to evaluate their applicability to complex environments.

One approach to overcome these limitations is to use acoustic metamaterials. Acoustic metamaterials are structures engineered to manipulate sound through either locally resonant or periodic structures [14]. Acoustic metamaterials can be designed to exhibit exotic properties, such as negative bulk modulus, negative mass density, and gradient refractive index, as well as sound manipulation functionalities such as sound focusing, beam steering, and acoustic pressure enhancement [15, 16, 17, 18, 19, 20]. Researchers are also implementing acoustic metamaterials to PZT‐based UEHs for enhanced efficiency and added functionalities [21, 22, 23]. For example, a study demonstrated a meta‐lens that focuses incoming ultrasound onto a PZT, thereby enhancing the energy harvesting performance [22]. Another study employed a metamaterial comprising a solid backing, a ring structure, and an air‐diffraction matrix to filter ultrasound frequencies [23]. Although these metamaterials improved UPT performances, they were solid structures that increased the weight and rigidity of UEHs, making them unsuitable for IMDs.

In this paper, we present a UEH enhanced with a flexible and lightweight metamaterial (Meta‐UEH) that overcomes the limitations of previously reported UEHs. The Meta‐UEH comprises a backing, a ring structure, and a PZT disc, all of which are encapsulated in a polydimethylsiloxane (PDMS) block. The backing and the ring structure induce local resonance at the PZT, which in turn increases its deformation and thereby increases the harvested electrical power. The main advantage of this approach is that the backing and the ring structure are composed of air, making them flexible, light, and immune to electrical failure. As a result, the Meta‐UEH achieves 350% of the harvested power of a UEH without the metamaterial, due to local resonance and the standing wave formed between the transducer and the metamaterial, while avoiding the reduced flexibility, added mass, and electrical complexity associated with prior UEHs employing PZT arrays and metamaterials.

## 2 Results

### 2.1 Design and Simulation of Meta‐UEH

**FIGURE 1:**
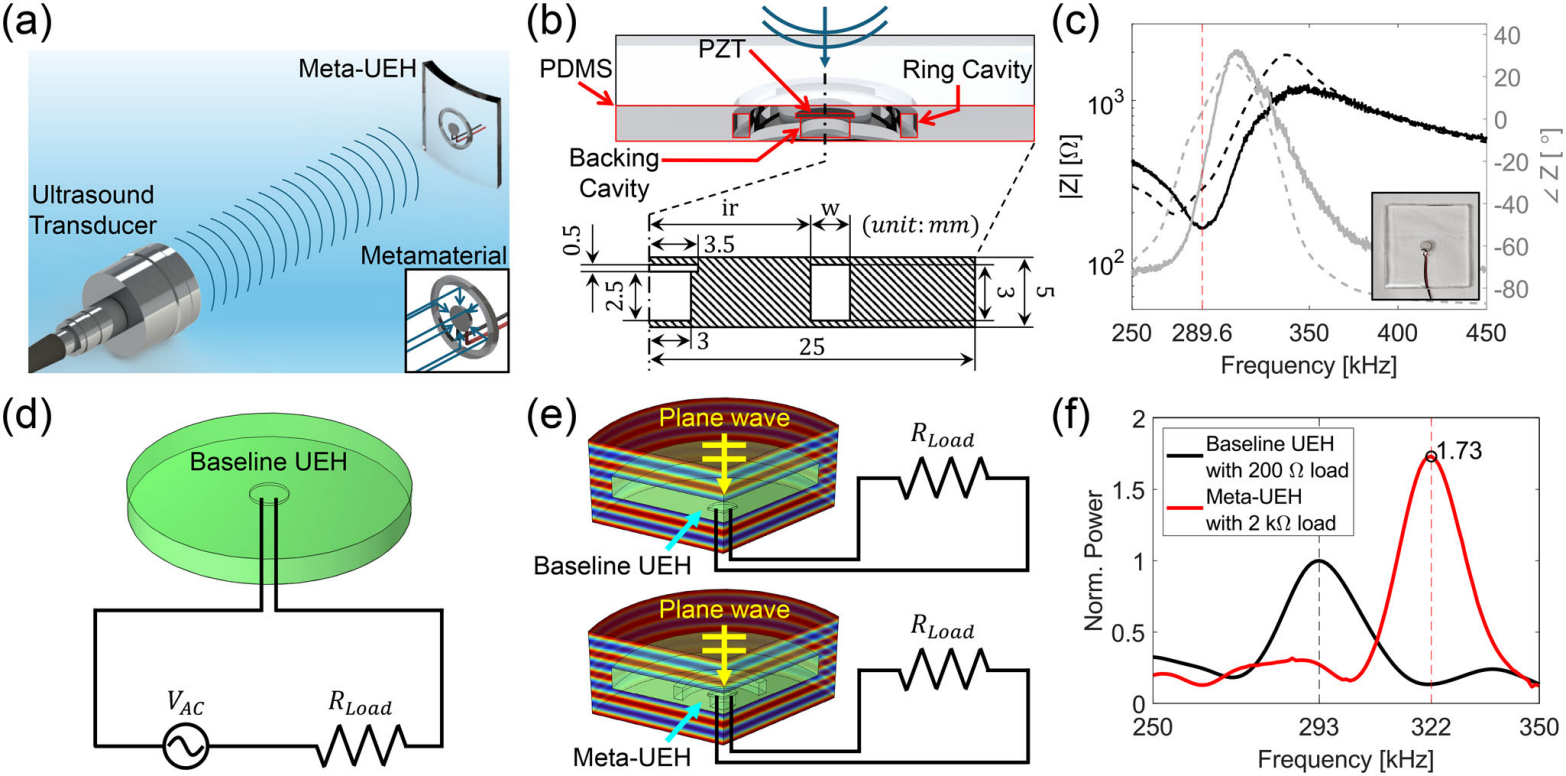
Design optimization and simulation of Meta‐UEH. (a) A schematic illustration of UPT from a transducer to a Meta‐UEH in water. The inset shows the metamaterial focusing the incoming acoustic energy onto the PZT. (b) Top: The cross‐section of the proposed Meta‐UEH showing its components. The outlines of the components on the cross‐section are highlighted with red lines. Bottom: An engineering drawing of the Meta‐UEH. The inner radius (ir) and width (w) of the ring structure were optimized for maximum energy harvesting performance. (c) The impedance of baseline UEH. Solid lines represent the measured impedance of the fabricated baseline UEH shown in the inset. Dashed black and gray lines are the simulated impedance of a baseline UEH model, and the red line marks the resonance frequency. (d) The simulation model used to obtain the impedance presented as dashed lines in Figure 1c. (e) Top: Simulation model of the baseline UEH under a plane wave. Bottom: Simulation model of the Meta‐UEH under a plane wave. Only one‐third of the models are illustrated to show the cross‐section. (f) Simulated performance of the baseline UEH with a 200 Ω load and the optimized Meta‐UEH with a 2 kΩ load.

As mentioned earlier, this work focuses on demonstrating the feasibility of using metamaterials to enhance the efficiency of UEHs while maintaining flexibility, lightweight, and minimal electrical components. Figure 1a shows our approach, in which an ultrasound transducer transmits ultrasound to a Meta‐UEH that harvests electrical energy. The blue lines between the transducer and the Meta‐UEH illustrate the transmitted ultrasound. Additionally, the inset depicts the concept of focusing the incoming acoustic energy onto the PZT at the center through local resonance.

The Meta‐UEH is composed of a PZT disc at the center, surrounded by an air‐filled backing and ring structure, all encased in a 5 cm × 5 cm × 5 mm PDMS block, as shown in the top panel of Figure 1b. This configuration positions the PZT at the forefront of the UEH, allowing the incoming ultrasound to interact directly with it and thereby ensuring maximum excitation. The backing is placed behind the PZT to reflect the ultrasound passing through the PZT back into it. The ring structure surrounding the backing allows the PDMS between them to resonate with the incoming ultrasound, thereby increasing PZT deformation. The engineering drawing in the bottom panel of Figure 1b presents the dimensions of Meta‐UEH components. Here, the inner radius (ir) and the width (w) of the ring structure are later optimized for maximum energy harvesting performance.

A PZT disc harvests electricity most efficiently when excited by sound that matches its resonance frequency [5]. Therefore, to set the operating frequency for this study, we measured the resonance frequency of a baseline UEH that features a PZT disc with a 7 mm diameter and 0.5 mm thickness at the center of the PDMS block without the metamaterial. The inset of Figure 1c is a photo of a fabricated baseline UEH. The impedance of the UEH, measured with an impedance analyzer, is presented in Figure 1c. The impedance graph clearly shows resonance occurring at 289.6 kHz (marked with a red dashed line) and an impedance magnitude of 159 Ω at the resonance.

Figure 1d illustrates a simulation model of the baseline UEH. Here, the model is circular, rather than rectangular, because the axisymmetric modeling approach was used to reduce computational load. To obtain the impedance, we connected the baseline UEH model in series with a 200 Ω load, which closely matches the measured impedance magnitude, and a 1V AC source. We then measured the voltage applied to both the load and the UEH and calculated the impedance using the following equation.

$$Z_{UEH}=\frac{V_{UEH}}{I}=\frac{V_{UEH}}{V_{Load}/R_{Load}}=R_{Load}\cdot \frac{V_{UEH}}{V_{Load}}$$

Here, $Z_{UEH}$, I, $R_{Load}$, $V_{UEH}$, and $V_{Load}$ represent the impedance of UEH, the current in the circuit, the resistance of the load, and the voltages applied to the UEH and the load, respectively. The dashed black and gray lines in Figure 1c show the impedance from the simulation model. The close agreement between the measured and simulated impedance confirms that the simulation model accurately represents the actual UEH. It also shows that using a circular geometry for axisymmetric modeling, instead of the actual square PDMS shape, has a negligible effect on the electrical characteristics of the PZT disc at the device center.

The simulation model was further used to estimate the energy harvesting performance of the baseline UEH. The top panel of Figure 1e shows the simulation setup, which involves a plane wave ensonifying the baseline UEH connected to a 200 Ω load. The frequency of the plane wave was swept from 250 to 350 kHz in 1 kHz increments. The power of the harvested electricity, calculated from the root mean square voltage ($V_{rms}$) according to $P=V_{rms}^{2}/R$, is presented in Figure 1f. This graph indicates that the resonance occurs at 293 kHz, which is similar to the resonance frequency measured from the impedance analysis.

To optimize the Meta‐UEH, the simulation model presented in the bottom panel of Figure 1e is used. The optimization process involved comparing the harvested power from the Meta‐UEH with a 200 Ω load while sweeping ir from 5 to 20 mm in increments of 1 mm and w from 1 to 3 mm in increments of 0.5 mm. From this process, we found that the combination of ir = 9 mm and w = 2 mm gives the best performance. Once these values were set, we swept the load from 10 Ω to 10 kΩ and found that the harvested power is largest when the load is 2 kΩ. Figure S1 provides the simulation results used for this optimization. The harvested power from the optimized Meta‐UEH with a 2 kΩ load is presented in Figure 1f. It is clearly shown that the metamaterial increases the power of the harvested electricity by 73%. In addition, the resonance frequency shifted to 322 kHz because, as discussed in subsection 2.3, the metamaterial altered the deformation behavior of the PZT by enhancing the radial displacement.

### 2.2 Fabrication and Experimental Demonstration of Meta‐UEH

**FIGURE 2:**
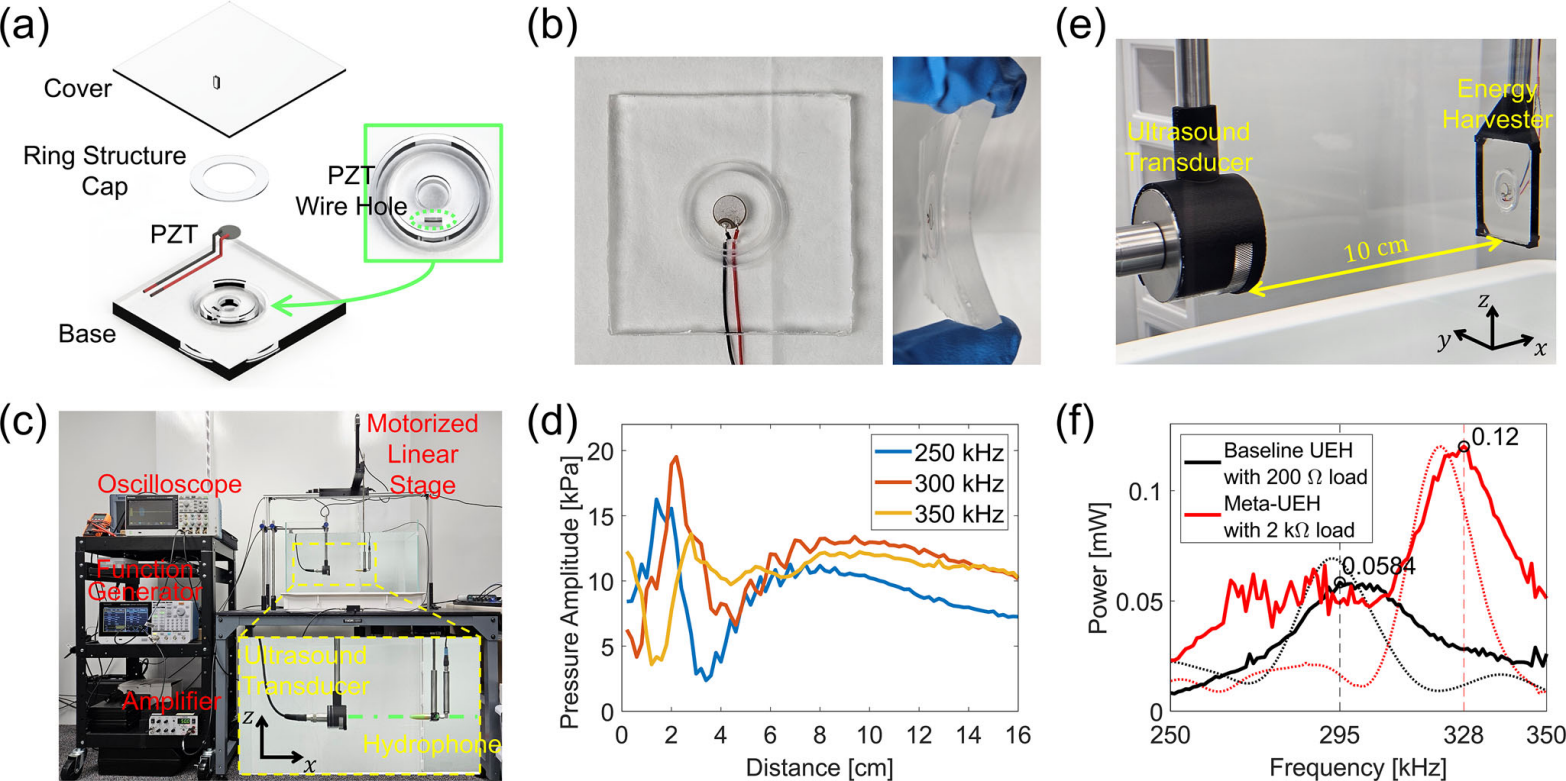
Fabrication and experimental demonstration of Meta‐UEH. (a) An exploded‐view drawing of the Meta‐UEH showing assembled parts. The inset shows a magnified view of the cavities and the hole in the base. (b) Pictures of the fabricated Meta‐UEH. (c) Measurement setup for characterizing the ultrasound transducer. The inset shows a magnified view of the transducer and hydrophone, along with a green dash‐dotted line indicating the transducer axis along which sound pressure measurements were taken at 2 mm intervals. (d) Measured pressure amplitude along the ultrasound transducer axis. (e) Experimental setup for demonstrating the Meta‐UEH. (f) Measured performance of the Meta‐UEH with a 2 kΩ load and the baseline UEH with a 200 Ω load. Dotted lines are simulation results from Figure 1f.

To experimentally demonstrate our approach, we fabricated the Meta‐UEH designed in the previous subsection. Figure 2a illustrates an exploded‐view drawing of the Meta‐UEH showing assembled parts. First, the base with cavities for the backing and ring structure, as well as a hole for PZT wires, was molded with PDMS. The inset in Figure 2a shows a magnified view of the elements. Once the base was prepared, we passed the PZT wires through the small wire hole and pasted the PZT and ring structure cap to the base using a very small amount of super glue. Once the PZT and ring structure cap are adhered, we molded the cover by pouring PDMS on top of the base‐PZT‐ring structure cap assembly. The left panel in Figure 2b presents the top view of the fabricated Meta‐UEH, and the right panel shows its side view when the device is bent to demonstrate its flexibility. The weight of the Meta‐UEH is 14 g, which is the same as that of the baseline UEH.

Before testing the Meta‐UEH, we characterized the ultrasound transducer used in this study. Figure 2c shows the ultrasound transducer and a hydrophone submerged in a water tank with dimensions of 59 × 34 × 32 ${\mathrm{cm}}^{3}$ (length × width × height). To determine the far field, where the transmitted ultrasound would have a plane wavefront similar to the incident wave used in the simulation, we measured the pressure amplitude of the transmitted ultrasound along the axis of the transducer, which is marked with a green dash‐dotted line in the inset of Figure 2c. For alignment, we first positioned the hydrophone so that its tip contacted the center of the transducer. We then used a motorized linear stage to move the hydrophone along the x‐axis, as marked in the inset of Figure 2c, in 2 mm steps. Here, the transducer was driven with a series of 10‐cycle burst signals, each with a 100 $V_{\text{pp}}$ amplitude and varying frequencies from 250 to 350 kHz in 50 kHz increments. Figure 2d presents the measured pressure amplitude along the transducer axis, clearly showing that the far field begins at a distance of 8 cm from the transducer.

For the experimental demonstration of the Meta‐UEH, we placed the sample 10 cm away from the transducer (Figure 2e) and transmitted a series of 10‐cycle bursts with frequencies from 250 to 350 kHz in 1 kHz increments. We note that 10‐cycle bursts were chosen to prevent standing waves due to reverberations between the transducer and the UEH. Longer bursts create spikes in the measurements caused by resonances from the reverberations, as shown in Figure S2. The experimental results are shown in Figure 2f, along with the simulation results from Figure 1f, which are represented by dotted lines for comparison. The measurements show that the harvested power from the Meta‐UEH is 205% of that from the baseline UEH, successfully validating the effectiveness of the metamaterial for enhancing UPT efficiency. The significant increase in power is even larger than the 73% improvement anticipated from the simulation. This discrepancy is due to the loss in the system, which reduces the quality factor and amplitude of the resonance. Although the performance of both UEHs degrades due to loss, Meta‐UEH is less affected because of its resonant characteristic. As a result, the losses present in the real system suppress the baseline UEH more than the Meta‐UEH, causing the experimentally measured enhancement to exceed the simulated expectation.

The reflectionless experimental setup used in this section differs from those in earlier studies on UEHs, in which the devices were tested with an ultrasound transducer directing waves onto a flat surface, producing reflections that formed a standing wave [7, 8, 9, 10, 11, 12, 13]. The strength of the standing wave depends on how flat the surface is, how parallel the surface and transducer are, and the distance between the transducer and the surface. By placing their UEHs inside strong standing waves, the studies achieved excellent energy harvesting performance. This experimental setup, however, is not realistic since the operating environments of UEHs would likely be surrounded by irregular boundaries that are neither flat nor parallel to the transducer. Therefore, the reflectionless setup presented here provides a conservative benchmark for evaluating UEH performance and can serve as a reference for future work.

### 2.3 Analysis and Evaluation of Meta‐UEH

**FIGURE 3:**
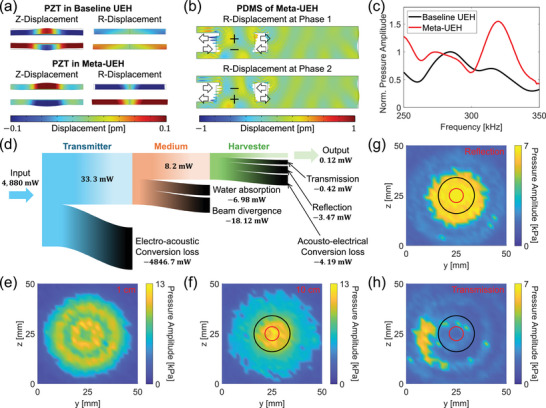
Working principle and energy flow visualization of Meta‐UEH. (a) The vertical and radial displacement of PZT in baseline UEH (top) and Meta‐UEH (bottom). (b) Radial displacement of the PDMS of Meta‐UEH at two different phases. The arrows indicate the displacement directions of the PDMS section between the backing and the ring structure. (c) Total acoustic pressure amplitude inside the PZT for baseline UEH and Meta‐UEH. (d) A Sankey diagram showing the energy flow of the Meta‐UEH. The links are not to scale for visual purposes. (e) Acoustic field measured at 1 cm from the ultrasound transducer. (f) Acoustic field measured at 10 cm from the ultrasound transducer. The red and black circles correspond to the areas covered by the 7 mm PZT disc diameter and the 9 mm ring structure inner diameter, respectively. (g) Reflected acoustic field measured 3.5 cm in front of the Meta‐UEH. (h) Transmitted acoustic field measured 3.5 cm behind the Meta‐UEH.

To understand the underlying mechanism of the energy harvesting performance improvement by the metamaterial, we analyzed the simulation model at its resonance frequency of 322 kHz. Figure 3a illustrates the displacement of PZTs inside the UEHs. The images reveal that the PZT in the Meta‐UEH deforms far more than the one in the baseline UEH in the radial (R) direction, while both PZTs deform at a similar magnitude in the vertical (Z) direction. This phenomenon is better visualized in the Video S1. To understand the increased R‐displacement for PZT in Meta‐UEH, we further analyzed the R‐displacement of PDMS. Figure 3b presents the R‐displacement of PDMS for Meta‐UEH at phases 0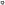 and 180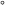, clearly showing that the region of PDMS between the backing and ring structure behaves like a vertical dipole cavity with amplified displacements in the R‐direction due to resonance. Here, we note that the vertical surfaces are not smooth due to the presence of surface waves.

The enhanced displacement responsible for the improved energy harvesting performance originates from the ring and backing air cavity. Owing to the large acoustic impedance mismatch between air and PDMS, the cavities form highly reflective boundaries that are geometrically focused toward the center of the ring where the PZT disk is positioned. This focusing effect, together with the trapping of acoustic energy between cavity boundaries, synergistically increases the sound pressure at the PZT, thereby enhancing energy harvesting efficiency. This effect is clearly shown by the total acoustic pressure amplitude inside the PZT, presented in Figure 3c, obtained by integrating absolute pressure amplitudes across the PZT.

To visualize the energy flow throughout the UPT process, we prepared a Sankey diagram (Figure 3d). The diagram consists of three sections, each representing the energy flow at the transmitter, medium, and harvester. In the transmitter section, the input to the system, which was the power of a 100 $V_{\text{pp}}$ AC signal at 300 kHz applied to the transducer, was measured to be 4.88 W. Then, as shown in Figure 3e, a 50 × 50 ${\mathrm{mm}}^{2}$ acoustic field of a 10‐cycle burst at 300 kHz across a plane 1 cm away from the transducer was scanned in a 2 mm grid to measure the acoustic power transmitted by the transducer. The acoustic power of this field, which was 33.3 mW, was calculated using the following equation.

$$\begin{array}{ccc} P & = & \int_{Z}\int_{Y}I\left(y,z\right)\;dy\;dz\approx \sum_{Z}\sum_{Y}I\left(y,z\right)\cdot A\\ & = & \sum_{Z}\sum_{Y}\frac{p_{\text{rms}}\left(y,z\right)}{\rho \cdot c}\cdot A=\sum_{Z}\sum_{Y}\frac{p_{amp}\left(y,z\right)}{\sqrt{2}\cdot \rho \cdot c}\cdot A \end{array}$$

Here, P, I, A, ρ, c, $p_{\text{rms}}$, and $p_{amp}$ represent the acoustic power, intensity, unit grid square area, water density, sound speed in water, root‐mean‐square acoustic pressure, and pressure amplitude, respectively. Once the power contained in the acoustic field was calculated, we subtracted it from the input power to the transducer and obtained the electro‐acoustic conversion loss.

Next, for the medium section, the energy absorbed by the water as sound propagated over the 10 cm distance from the transducer to the Meta‐UEH was calculated by subtracting the power contained in the acoustic field at 10 cm (Figure 3f), which was 26.32 mW, from the power in the field at 1 cm. In Figure 3f, the red circle represents the area corresponding to the 7 mm diameter PZT at the center of the Meta‐UEH, and the black circle represents the area covered by the inner radius of the ring structure. To calculate the beam divergence, we summed the acoustic energy outside of the black circle and obtained 18.12 mW. Additionally, the energy passed onto the harvester was calculated by summing the acoustic energy inside the black circle, which was 8.2 mW.

Subsequently, for the harvester section, we measured the reflection and transmission of the Meta‐UEH by scanning the acoustic fields on 2 mm grids at locations 3.5 cm in front of and behind the Meta‐UEH (Figure S3) [24]. The measurements showed that the reflected and transmitted power within the black circles in Figure 3g,h were 3.47 and 0.42 mW, respectively. Finally, the acousto‐electrical conversion loss, which includes thermal energy loss from repeated oscillation of PDMS and inherent mechanical‐to‐electrical conversion loss in PZT, was calculated by subtracting the output power (0.12 mW), reflection, and transmission from the acoustic power of 8.2 mW that arrived at the black‐circled area.

The diagram reveals that the port‐to‐port power transfer efficiency is 0.0025%, with the electro‐acoustic conversion loss accounting for 99.3% of the total loss. If we exclude the loss from the transmitter, the efficiency becomes 0.36%, and the most significant contributor to the loss is the beam divergence, accounting for 54.41%.

We note that the 0.0025% or 0.36% power transfer efficiency is lower than the typical 3 ∼ 35% efficiency achieved by PZT‐based UEHs in other studies [5, 25, 26, 27, 28, 29, 30]. One reason for this is that the transducer was not operating at its designed resonance frequency of 500 kHz. The energy efficiency of a transducer is highest at its resonance frequency and decreases as the frequency moves away from this point (Figure S4a–c). The frequency range used in this study was nearly one octave lower than the resonance frequency of the transducer, and thus caused 99.3% of the energy loss. Another reason is that the UEHs used in other studies were typically designed to be larger or of equal size to the transducer, allowing them to capture most of the acoustic energy generated by the transducer (Figure S4d–f). In our case, the diameter of the transducer and the PZT were 44 and 7 mm, respectively, resulting in an excessive beam divergence loss. This could be reduced by using a transducer that focuses ultrasound onto the PZT, which was beyond the scope of this study. Lastly, as previously explained, using a reflectionless setup produced a conservative result. Other studies adjusted the distance between the transducer, the UEH, and the background floor so that the continuous ultrasound would resonate between them. In our experiment, on the other hand, we specifically avoided resonance by separating the transducer and the UEH with a large distance and using short bursts. In the following subsection, it is demonstrated that the harvested power increases by a factor of 4.2 when we induce resonance between the transducer and the UEH by implementing continuous waves. As a result, under ideal conditions, including negligible transducer loss, no beam divergence, and strong resonance between the transducer and the UEH, the efficiency would increase to 19.15%. Although this value is comparable to those reported in other studies, it was achieved using a considerably smaller PZT disc with a 7 mm diameter and 0.5 mm thickness.

### 2.4 Demonstration of Meta‐UEH Under Reverberation and Deformation

**FIGURE 4:**
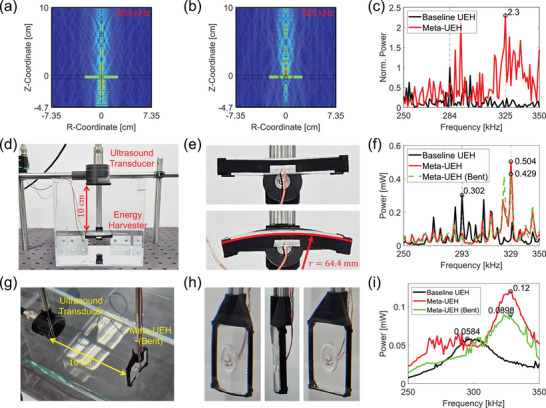
Demonstration of Meta‐UEH under continuous wave in a reflective environment. (a,b) Simulated acoustic fields under continuous waves in a small water cylinder surrounded by reflective walls for (a) baseline UEH with 284 kHz ultrasound, and (b) Meta‐UEH with 325 kHz ultrasound. (c) Simulation results for the UEHs in the 250 ∼ 350 kHz frequency range. (d) Experimental setup for testing the UEHs in a small water container with continuous waves. (e) Top: Meta‐UEH in natural configuration. Bottom: Meta‐UEH in bent configuration. (f) Measured harvested power from the UEHs. (g) Experimental setup for evaluating the effect of air cavity deformation on Meta‐UEH performance. (h) Left: Meta‐UEH in bent configuration. Center: Side view of the bent Meta‐UEH. Right: Meta‐UEH in natural configuration shown for reference. (i) Meta‐UEH performance in the bent configuration in a reflectionless setup. Performance of the baseline UEH and the Meta‐UEH in the natural configuration from Figure 2f is also shown for comparison.

To validate the robustness of the Meta‐UEH in the presence of reflections, we tested the UEHs in a smaller water container with continuous waves. Figure 4a,b illustrates our simulation model along with the resulting pressure fields at 284 and 325 kHz, respectively. In the model, a water cylinder with a diameter and height of 14.7 cm was used. The water was surrounded by reflective hard boundaries, and a UEH was placed 10 cm below the water surface. Additionally, an ultrasound source with a diameter of 44 mm was placed at the center of the surface, emitting a series of continuous waves with frequencies ranging from 250 to 350 kHz in 1 kHz increments. The acoustic fields show that more energy is concentrated between the UEH and the water surface for Meta‐UEH than for baseline UEH. This is because the Meta‐UEH is more reflective than the baseline UEH, resulting in stronger standing waves. For baseline UEH, on the other hand, more acoustic energy penetrates through it and reaches the bottom surface. This is shown in both Figure 4a and Figure S5.

Figure 4c presents the simulated harvested power from the models. The graph shows that Meta‐UEH continues to harvest more power than baseline UEH even in the presence of reflections. Additionally, the maximum harvesting performance is achieved at 325 kHz, which is similar to the resonance frequency of the Meta‐UEH (322 kHz). However, the plot is noisy over the frequency range because the position of the UEH becomes the node and anti‐node depending on the wavelength.

To experimentally validate this observation, we used the setup shown in Figure 4d. A 14.7 × 14.7 × 14.7 ${\mathrm{cm}}^{3}$ water container was prepared, and the Meta‐UEH was positioned 10 cm below the surface. An ultrasound transducer was placed at the water surface to transmit continuous wave signals swept over the same frequency range as in the simulation. The Meta‐UEH was tested in two configurations, namely the natural position and bent position, as shown in Figure 4e, to evaluate its performance under deformation.

Figure 4f is the measured harvested power from the baseline UEH and Meta‐UEH. The graph confirms that Meta‐UEH maintains improved energy harvesting performance compared to the baseline UEH, resulting in a 66.9% power increase, even in a reflective environment with continuous waves. Additionally, the Meta‐UEH in bent configuration continued to perform better than the baseline UEH, although it was not as effective as it was in the natural configuration. To ensure the performance improvement is not specific to the 10 cm distance, we conducted the same test at 5 and 3 cm as well and confirmed the performance improvement at other locations (Figure S6).

One reason for the decreased harvested power in the bent configuration is the reduced standing wave strength. For continuous waves, energy harvesting performance not only depends on the local resonance from the metamaterial, but also on the resonance of the standing wave between the transducer and the UEH. The reflections from the PDMS surface are more directed toward the transducer when the Meta‐UEH is at its natural configuration, resulting in the maximum reverberation and thereby a stronger standing wave. When the Meta‐UEH is bent, on the other hand, the reflection diverges, and only a fraction of the reflected energy reaches the transducer, thereby reducing the energy contained in the standing wave. It should be noted, however, that this is not the case when the distance between the transducer and Meta‐UEH is short, and thus, most of the reflections arrive at the transducer despite the divergence. This explains why the performance of the bent configuration does not decrease compared to that of the natural configuration when the distance is 3 cm, as shown in Figure S6c.

Another reason for the reduced performance is the deformation of the air cavity when the Meta‐UEH is bent. To isolate the effect of this deformation, we tested the bent Meta‐UEH in the reflectionless setup we used in Section 2.2, where the ultrasound transducer and the device were placed 10 cm apart inside the water tank (Figure 4g). For consistency, we used 10‐cycle bursts at frequencies from 250 to 350 kHz in 1 kHz increments. Figure 4h provides close‐up images of the Meta‐UEH mounted in its bent configuration. The result, presented in Figure 4i, confirms that the deformation of the air cavities reduces harvested power at the resonance frequency from 0.12 to 0.0898 mW. This finding indicates that for applications where the Meta‐UEH is expected to undergo significant deformation, its structure should be redesigned and optimized for the bent state to avoid a substantial degradation in performance.

The harvested power of 0.504 mW from the Meta‐UEH in the small water container under continuous waves at a 10 cm distance from the transducer is much smaller than the typical 21 ∼ 72 mW output power values reported in other studies on PZT‐based UEHs [5, 25, 26, 27, 28, 29, 30]. This is because the sound amplitude of $p_{amp}=$ 13 kPa that excited the UEH at the 10 cm location shown in Figure 2d is much smaller than the highest amplitude allowed for the human body, which is 146.97 kPa at 300 kHz if we consider the spatial peak temporal average intensity (ISPTA) limit of 720 mW/${\mathrm{cm}}^{2}$ set by the U.S. Food and Drug Administration [31, 32, 33] (Derivation is provided in Note S1) or the amplitudes used in other studies. We were unable to use the highest amplitude because, as mentioned earlier, we were not operating the transducer at its designed resonance frequency of 500 kHz but at the resonance frequency of the PZT disc, which limited us to producing a smaller amplitude before clipping occurred. If we ensonify the UEH with the maximum ultrasound amplitude allowed and assume the UPT system to be linear, the harvested voltage amplitude is expected to increase by 146.97/13 = 11.3 times. Consequently, the harvested power would increase by 11.3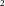 = 127.69 times, giving 0.504 × 127.69 = 64.4 mW, which is a value that is comparable to previously reported results [5]. This calculation was further validated through numerical simulation, with the result shown in Figure S7. Additionally, if we follow [27] and use a power density of 94 mW/${\mathrm{cm}}^{2}$, obtained by generating a standing wave between two 15 mm diameter PZT transducers separated by a 5 cm gap, the harvested power would increase to 8.41 mW. However, the scope of this study was limited to validating the effectiveness of metamaterials for enhancing UPT by demonstrating the improved energy harvesting performance of the Meta‐UEH compared to the baseline UEH. Therefore, instead of developing an optimized transducer for maximum harvested power, we used a commercially available transducer.

## 3 Conclusion

In this study, we demonstrated the effectiveness of utilizing metamaterials to enhance the energy harvesting performance of UEHs. The proposed Meta‐UEH is flexible, light, and composed of minimal electrical components, making it suitable for IMDs. The device was first evaluated in a reflectionless setting, both experimentally and through simulation, and it demonstrated improved energy harvesting performance, achieving up to 205% of the harvested power of a baseline UEH. To understand the origin of the improvement, we analyzed the displacements occurring in the Meta‐UEH. From the analysis, we discovered that the section of PDMS between the backing and the ring structure acted as a resonant cavity, increasing the deformation of the PZT in the radial direction. This suggests that the improvement from the metamaterial is not specific to PZTs and can be applied to other acoustic‐electric energy conversion mechanisms, such as PENGs and TENGs, whose performance depends on the magnitude of deformation. Additionally, a Sankey diagram was prepared to identify the energy loss at each stage, setting a benchmark for future studies. Finally, the robustness of the Meta‐UEH to reflections and deformations was tested in a small water container with continuous waves, successfully outperforming the baseline UEH under reverberations in a bent configuration. Remarkably, the harvested power from the Meta‐UEH under reverberations increased by both the local resonance and the resonance between the transducer and the metamaterial, reaching up to 350% of that from a baseline UEH without resonance. This work presents significant potential for addressing the limitations of IMDs related to battery capacity and power usage through Meta‐UEHs while establishing guidelines for designing, testing, and evaluating implantable metamaterials for UPT.

## 4 Experimental Section

### 4.1 UEH Fabrication

As shown in Figure 2a, Meta‐UEH was composed of four components, namely the base, ring structure cap, PZT, and cover. First, the base and ring structure cap were fabricated by casting PDMS into 3D‐printed molds. For PDMS, we used the Sylgard 184 Silicone Elastomer Kit from Dow Chemical Company. We mixed the elastomer base and the curing agent in a 10:1 ratio and cured it at 23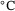 for 72 h. Here, 23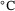 was chosen to prevent the air cavities from deforming due to the temperature difference between the curing process and the experimental setup.

Once the PDMS is cured, a wired PZT disc and the ring structure cap were pasted to the base. The PZT disc was an SMD07T05R412WL piezoelectric ceramic disc from STEMINC PIEZO. It is composed of SM412 piezo material with a static capacitance of 2.5 nF and thickness direction polarization. For pasting, we used Super Glue from Elmer's.

To seal the base‐PZT‐ring structure cap assembly with the cover, we placed the assembly at the bottom of a mold and poured PDMS on top of it. For the Baseline UEH, the process was identical to that of Meta‐UEH without the backing and ring structure.

### 4.2 Baseline UEH Characterization

To characterize the impedance and resonance frequency of the baseline UEH, an Analog Discovery 3 portable measurement unit from Digilent was used. The baseline UEH was connected to the Analog Discovery 3 with an Impedance Analyzer adapter accessory, and WaveForms version 3.23.3 software was used to measure its impedance in the 250 ∼ 450 kHz frequency range.

### 4.3 Experimental Demonstration of UEH

To demonstrate the energy harvesting performance of the UEHs, we used an experimental setup shown in Figure 2c. Here, a Tektronix AFG31102 arbitrary function generator was used to produce a series of 5 $V_{\text{pp}}$ amplitude 10‐cycle bursts with 20 ms trigger delay. The bursts were then amplified to 100 $V_{\text{pp}}$ by a Krohn–Hite 7602M wideband power amplifier and transferred to an Olympus V389‐SU ultrasound transducer.

To characterize the transducer, and thus identify its near field and far field, an Onda HGL‐0400 capsule hydrophone with an AG‐2010 preamplifier was used. The hydrophone was attached to three Zaber X‐LSM200A motorized linear stages, which moved the hydrophone along the transducer axis with a 2mm step. The measurements from the hydrophone were read using a Tektronix TBS2074 digital oscilloscope. In this experiment, the function generator, motorized stages, and oscilloscope were connected to a desktop to automate the experiment using MATLAB.

Once the far field was identified, the hydrophone was replaced by a UEH, as presented in Figure 2e. When demonstrating the UEHs, resistors matching their impedance were connected to maximize the power of the harvested electricity, which were 200 Ω for the baseline UEH and 2 kΩ for the Meta‐UEH.

### 4.4 Power Measurements

To measure the electrical power applied to the ultrasound transducer, we used a Tektronix TCP0020 current probe. Specifically, the current probe was clipped to a wire connecting the amplifier and the transducer. Then, the current measurement, which was 750 ${\mathrm{mA}}_{\text{pp}}$, was multiplied by the voltage transmitted from the function generator to the amplifier, giving an outcome of 244 mW. Assuming the amplifier increases the voltage from 5 $V_{\text{pp}}$ to 100 $V_{\text{pp}}$ linearly and without additional phase delay, the input power to the transducer from the amplifier was calculated as 244 × 20 = 4,880 mW.

For measuring the acoustic power produced by the transducer at 1 and 10 cm plane, as shown in Figure 3e,f, we used Onda HNR1000 needle hydrophone. The hydrophone was attached to the motorized stages to scan the 5 × 5 ${\mathrm{cm}}^{2}$ fields with a 2 mm step. Then, the measurement amplitudes in voltage were converted to pressure amplitudes using the calibrated sensitivity value provided by the manufacturer.

### 4.5 Simulation of UEH

The behaviors of the UEHs were simulated using Comsol Multiphysics. As illustrated in Figure 1e, the finite element models were developed for simulation in 2D Axisymmetric space. They contained the Pressure Acoustics module, the Solid Mechanics module, the Electrostatics module, and the Electrical Circuit module, all in the frequency domain. The dimensions of the UEHs were the same as presented in Figure 1b. For background, we used a 10.5 mm height and a 6 cm diameter, with edges set to be perfectly matched boundaries for simulation in a reflectionless environment, and a 14.7 cm height and a 14.7 cm diameter, with edges set to be sound hard boundaries for simulation in a reflective environment.

For PDMS, its density, pressure‐wave speed, shear‐wave speed, and isotropic structural loss factor were set to 1026.15 kg/$m^{3}$, 1090 m/s, 152 m/s, 0.1, respectively. For water, air, and PZT, we used built‐in materials stored in the material library of Comsol Multiphysics. Here, for PZT, we used Lead Zirconate Titanate (PZT‐5A).

## Conflicts of Interest

The authors declare no conflicts of interest.

## Supporting information


**Supporting File**: adma72751‐sup‐0001‐SuppMat.pdf.

**Supporting File**: adma72751‐sup‐0002‐VideoS1.gif.

## Data Availability

The data that support the findings of this study are available from the corresponding author upon reasonable request.
