# Supplementary material for: An Energy Focusing Flexible and Lightweight Acoustic Metamaterial for Enhanced Ultrasound Power Transfer
Source: Adv Mater. 2026 Mar 11;38(20):e19545. doi: 10.1002/adma.202519545 (PMC13054120; doi:10.1002/adma.202519545)
Supplement: Supplementary file 1 — Supporting File: adma72751‐sup‐0001‐SuppMat.pdf. [file ADMA-38-e19545-s001.pdf]

Supporting Information for

An Energy Focusing Flexible and Lightweight Acoustic Metamaterial for Enhanced Ultrasound Power Transfer

Hyung-Suk Kwon, Ziqi Yu, Xiaopeng Li, Ercan M. Dede, and Taehwa Lee**

H.-S. Kwon, Z. Yu, X. Li, E. M. Dede, T. Lee

Electronics Research Department,

Toyota Research Institute of North America,

Ann Arbor, MI 48105, USA

E-mail: hyung.suk.kwon@toyota.com; taehwa.lee@toyota.com

Supporting information included in this file:

- Supporting Figure S1 – S7
- Supporting Note S1

Other supporting information:

- Supporting Video S1

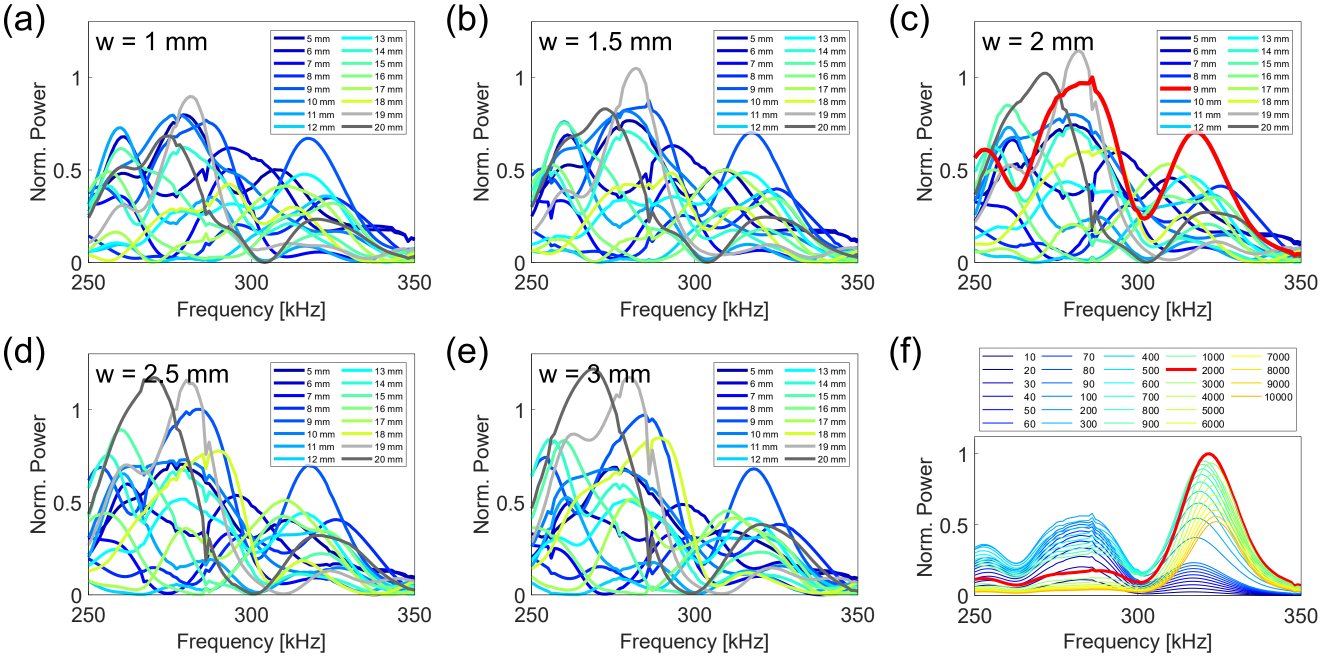

Figure S1. Simulation results used for Meta-UEH optimization. (a)-(e) Harvested power from the Meta-UEH with a 200 Ω load as the ring structure width (*w*) is varied from 1 mm to 3 mm in increments of 0.5 mm, and the inner radius (*ir*) from 5 mm to 20 mm in increments of 1 mm. Although inner radii of 19 mm and 20 mm occasionally exhibit higher peak performance than the selected parameters (*w* = 2 mm and *ir* = 9 mm; thick red line in Figure S1c), this enhancement is limited to a narrow frequency range, whereas the selected design provides strong energy harvesting performance over a broader frequency range, making it a more robust choice. (f) Harvested power from the Meta-UEH with the selected parameters (*w* = 2 mm and *ir* = 9 mm) for load resistances ranging from 10 Ω to 10 kΩ. The 2 kΩ load (thick red line) yields the highest harvested power.

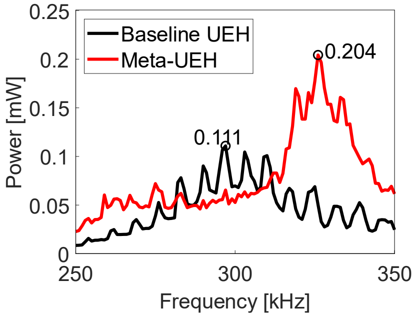

Figure S2. Measurements with resonance peaks due to reverberations between the transducer and UEH when 70 peak bursts are used.

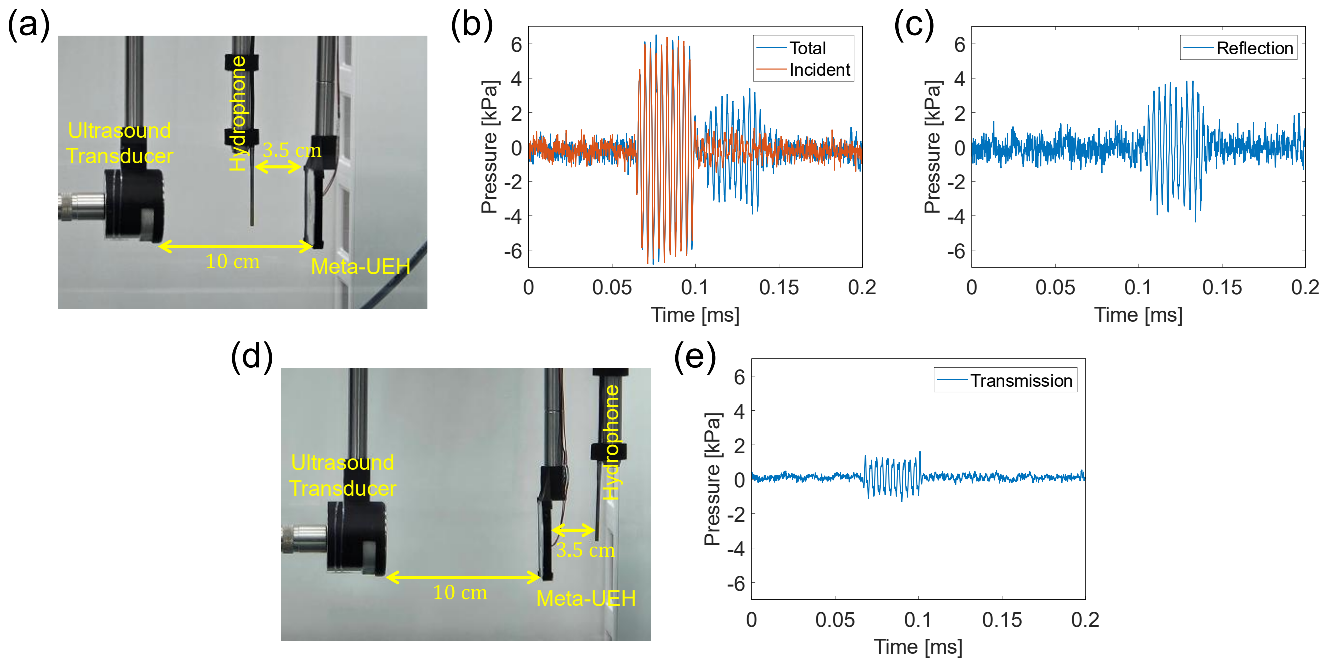

Figure S3. Reflection and transmission measurement setup and sample data. (a) Experimental setup for reflection measurements. (b) Time domain signal measured when the hydrophone tip is positioned at the center, 3.5 cm in front of the Meta-UEH. Total denotes the measurement made with the Meta-UEH in the frame, while Incident denotes the measurement made without the Meta-UEH. (c) Reflected signal obtained by subtracting the Incident signal from the Total signal. (d) Experimental setup for transmission measurements. (e) Time-domain signal measured when the hydrophone tip is positioned at the center, 3.5 cm behind the Meta-UEH.

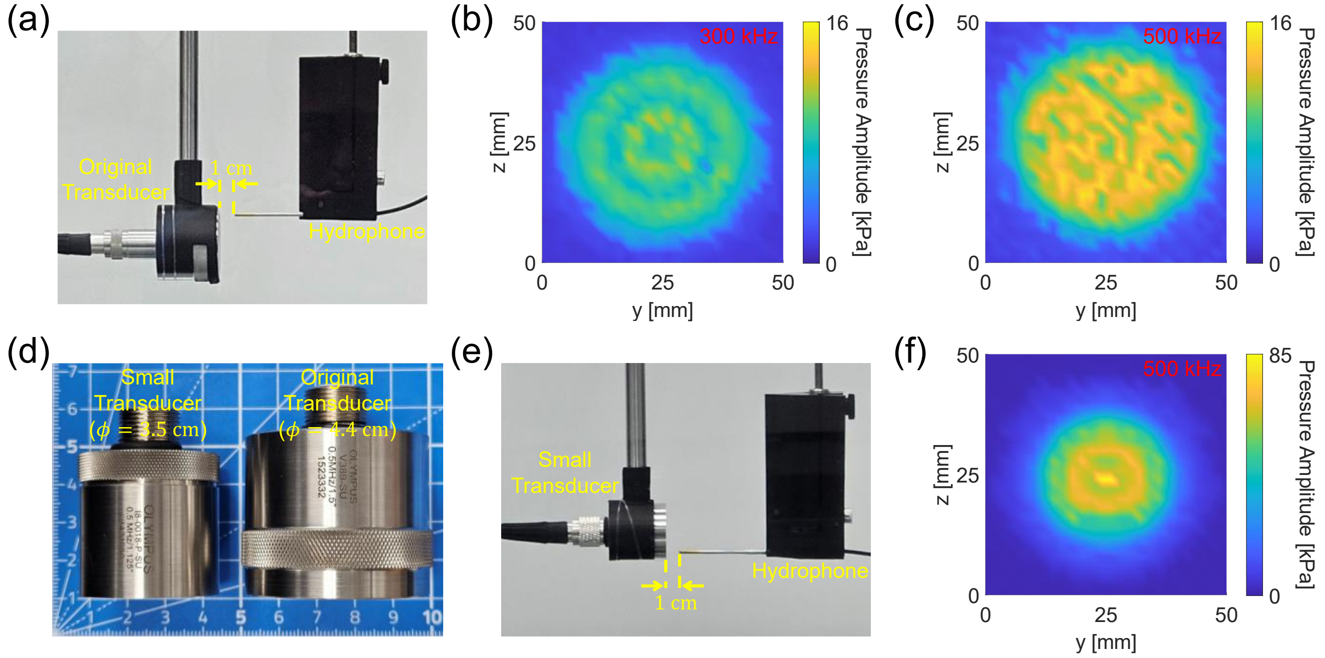

Figure S4. Effects of transducer resonance frequency and diameter on energy conversion efficiency. (a) Experimental setup for measuring the acoustic power produced by the transducer on a 5 cm × 5 cm plane located 1 cm from the transducer surface. (b) Measured acoustic field at 1 cm for a 300 kHz, 10-cycle burst. This measurement corresponds to Figure 3e, shown using a different color scale for consistency. (c) Measured acoustic field at 1 cm for a 500 kHz, 10-cycle burst. The acoustic power in this field is 64.07 mW, which is 1.9 times larger than the 33.3 mW obtained at 300 kHz. This result indicates that the transducer efficiency is significantly reduced when the device is operated below its designed resonance frequency. (d) Photograph of the original transducer and a smaller transducer (Olympus I8-0018-P-SU) prepared to examine the effect of transducer diameter on energy conversion efficiency. The smaller transducer has a diameter of 3.5 cm and a resonance frequency of 500 kHz. (e) Experimental setup for measuring the acoustic power produced by the smaller transducer on a plane located 1 cm from the transducer surface. The transducer was driven with 100 V_{pp}, 10-cycle bursts to maintain consistency with other experiments. (f) Measured acoustic field from the smaller transducer at 1 cm for a 500 kHz, 10-cycle burst. The acoustic power in this field is 670.41 mW, which is substantially larger than that of the original transducer because nearly all of the acoustic energy is confined within the scanning area.

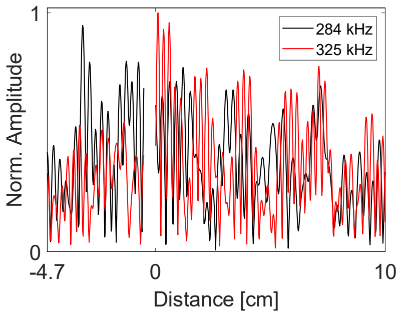

Figure S5. Pressure along the z-axis for the acoustic pressure fields illustrated in Figure 4a and 4b.

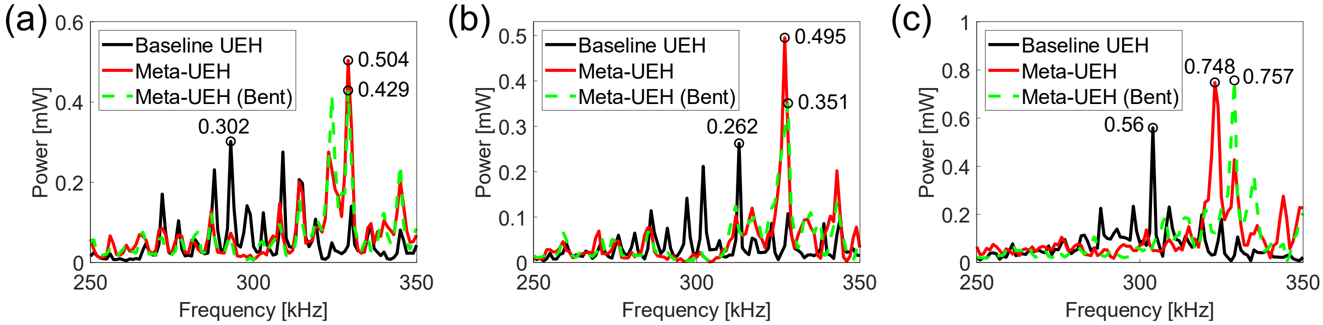

Figure S6. Energy harvesting in a small water container when the distance between the transducer and UEHs is (a) 10 cm, (b) 5 cm, and (c) 3 cm.

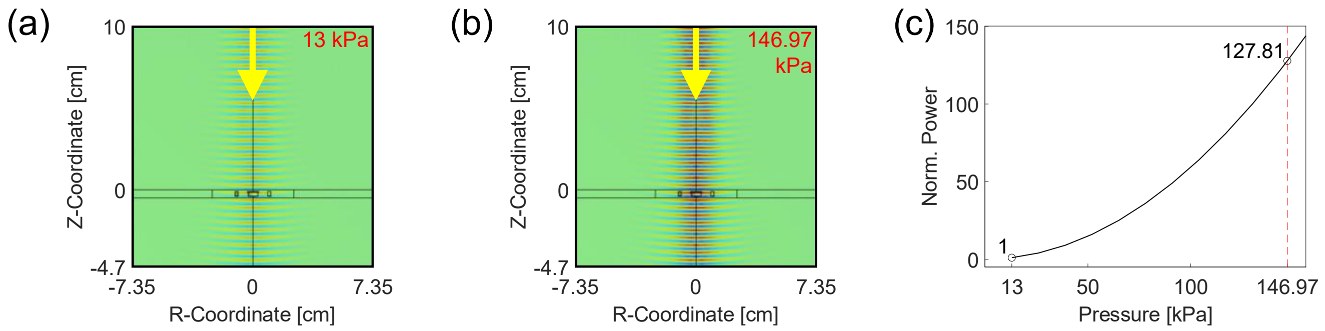

Figure S7. Numerical simulation validating the calculated increase in harvested power under the maximum allowed ultrasound amplitude. (a) Incident ultrasound with 13 kPa amplitude, as used in the experiment. (b) Incident ultrasound with 146.97 kPa amplitude, the FDA limit. (c) Simulated harvested power as a function of incident ultrasound amplitude, normalized to the harvested power at 13 kPa. The simulation predicts a 127.81-fold increase from 13 kPa to 146.97 kPa, closely matching the calculated value of 127.69-fold.

Note S1. Derivation of the maximum sound amplitude allowed in the human body.

The Food and Drug Administration limits the spatial peak temporal average (SPTA) intensity allowed in the human body to $720\ \mathrm{mW/cm^2}$ ($= 7200\ \mathrm{W/m^2}$) [1-3]. The intensity is calculated using the following equation.

$$\mathrm{I_{SPTA}} = \frac{p_{\mathrm{rms}}^2}{\rho \cdot c}$$

Here, $\mathrm{I_{SPTA}}$, p_{rms}, ρ, c are SPTA intensity, root-mean-square (RMS) sound pressure, density of the medium, and sound speed in the medium, respectively.

We used water as the medium in our experiment. Therefore, the density and sound speed are $\rho_{\mathrm{water}} = 1000\,\mathrm{kg/m^3}$ and $c_{\mathrm{water}} = 1500\,\mathrm{m/s}$.

From this, the RMS pressure can be calculated as

$p_{\mathrm{rms}} = \sqrt{\rho \cdot c \cdot \mathrm{I_{SPTA}}} = \sqrt{1000 \cdot 1500 \cdot 7200} = 103.92$ kPa.

Therefore, the maximum amplitude of the sound pressure allowed in our experiment is $p = \sqrt{2} \cdot p_{\mathrm{rms}} = \sqrt{2} \cdot 103.92 = 146.97$ kPa.

References

[1] Food and Drug Administration, Guidance for Industry and Food and Drug Administration Staff. Available online: https://www.fda.gov/regulatory-information/search-fda-guidance-documents/marketing-clearance-diagnostic-ultrasound-systems-and-transducers (accessed on 26 August 2025) 2019.

[2] T. R. Nelson, J. B. Fowlkes, J. S. Abramowicz, C. C. Church 2009.

[3] C. M. I. Quarato, D. Lacedonia, M. Salvemini, G. Tuccari, G. Mastrodonato, R. Villani, L. A. Fiore, G. Scioscia, A. Mirijello, A. Saponara, et al., Diagnostics 2023, 13, 5 855.
