# Supplementary figures and images for: An Energy Focusing Flexible and Lightweight Acoustic Metamaterial for Enhanced Ultrasound Power Transfer
Source: Adv Mater. 2026 Mar 11;38(20):e19545. doi: 10.1002/adma.202519545 (PMC13054120; doi:10.1002/adma.202519545)

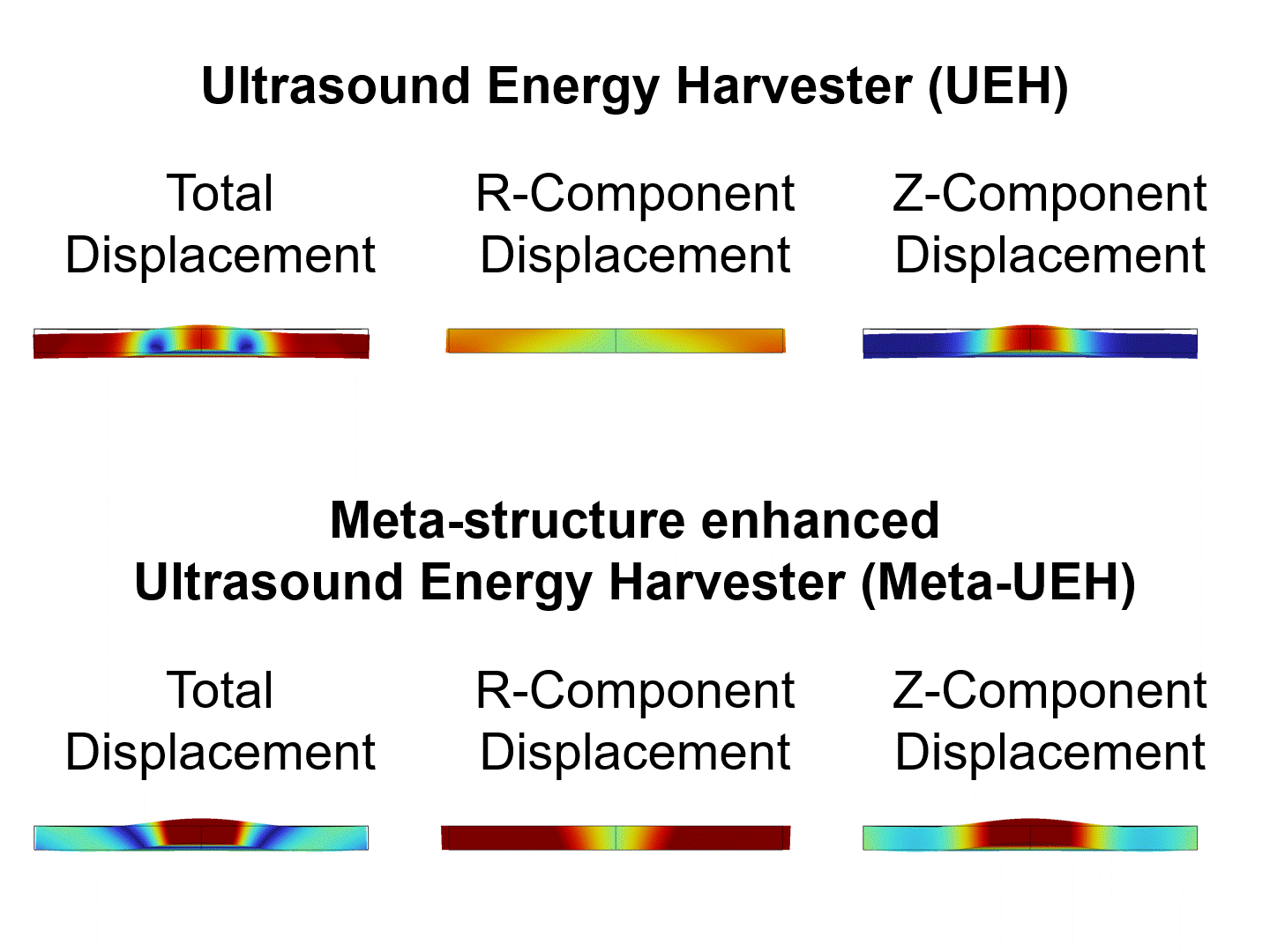

Supplement: Supplementary file 2 — Supporting File: adma72751‐sup‐0002‐VideoS1.gif. [file ADMA-38-e19545-s002.gif]
